# Inflammasomes and Pyroptosis of Liver Cells in Liver Fibrosis

**DOI:** 10.3389/fimmu.2022.896473

**Published:** 2022-05-30

**Authors:** Can Gan, Qiuyu Cai, Chengwei Tang, Jinhang Gao

**Affiliations:** ^1^ Lab of Gastroenterology and Hepatology, West China Hospital, Sichuan University, Chengdu, China; ^2^ Department of Gastroenterology, West China Hospital, Sichuan University, Chengdu, China

**Keywords:** inflammasome activation, pyroptosis, caspase-1, IL-1β, IL-18, liver cirrhosis

## Abstract

Inflammasomes are multiprotein complexes that can sense danger signals and activate caspase-1 to mediate pro-inflammatory cytokines release and pyroptotic cell death. There are two main canonical and non-canonical signaling pathways that trigger inflammasome activation. Inflammasomes are expressed and assembled in parenchymal and nonparenchymal cells in response to liver injury in the liver. Additionally, the hepatocytes, biliary epithelial cells (cholangiocytes), hepatic stellate cells (HSCs), hepatic macrophages, and liver sinusoidal endothelial cells (LSECs) contribute to liver fibrosis *via* different mechanisms. However, the underlying mechanism of the inflammasome and pyroptosis in these liver cells in liver fibrosis remains elusive. This review summarizes the activation and function of inflammasome complexes and then discusses the association between inflammasomes, pyroptosis, and liver fibrosis. Unlike other similar reviewers, we will focus on the effect of inflammasome activation and pyroptosis in the various liver cells during the development of liver fibrosis. We will also highlight the latest progress of pharmacological intervention in inflammasome-mediated liver fibrosis.

## 1 Introduction

Inflammasomes are signaling platforms in response to infectious diseases or chronic sterile inflammation. These multimeric complexes respond to molecular patterns from pathogens and cellular damage by releasing pro-inflammatory cytokines and inducing pyroptotic cell death (1). In general, inflammasome complexes are composed of a pattern-recognition receptor, an effector caspase-1, and an adaptor connecting these two components. There are two groups of inflammasomes in terms of receptors: the NLR family containing NLRP1, 2, 3, 6, NLRC4 and NLRP12; pyrin and HIN domain-containing (PYHIN) family including absent in melanoma 2 (AIM2) and pyrin (2). The inflammasomes could either aggravate inflammation *via* interleukin (IL)-1β and IL-18 or induce pyroptosis *via* gasdermin D (GSDMD). Although the inflammasome and its related pyroptosis have been well studied in infectious diseases, the role and mechanism of inflammasome and pyroptosis in liver fibrosis remain unclear.

Liver fibrosis is characterized by excessive extracellular matrix (ECM) deposition in response to chronic liver injury, including virus infection, non-alcoholic steatohepatitis (NASH), alcoholic liver disease (ALD), and autoimmune diseases (3, 4). Generally, activated HSCs are primary myofibroblasts that produce and secrete ECM. In addition, hepatocytes, Kupffer cells (KCs), LSECs, cholangiocytes, and recruited cell types (*e.g.*, bone marrow-derived macrophages) also contribute to liver fibrosis (5, 6). The mechanisms of liver fibrosis are complicated and involve different cells, signaling pathways, and cross-talk between individual cells. Recent studies have shown that inflammasomes and inflammasome-related pyroptosis are involved in liver fibrogenesis from various pathologies (7). The detailed mechanism of the inflammasome and pyroptosis in liver fibrosis is not well-defined. Collaboration of multiple liver cells maintains hepatic homeostasis in health and contributes to disturbed hepatic homeostasis in liver fibrosis (8). This review summarizes the progress of inflammasome and inflammasome-related pyroptosis in liver fibrosis. The roles and mechanisms of inflammasomes and pyroptosis in various liver cells and their cross-talks will also be described. Eventually, the potential therapeutic targets and future directions will also be implied based on the current progress.

## 2 Inflammasome Biology and Activation

**Figure 1:**
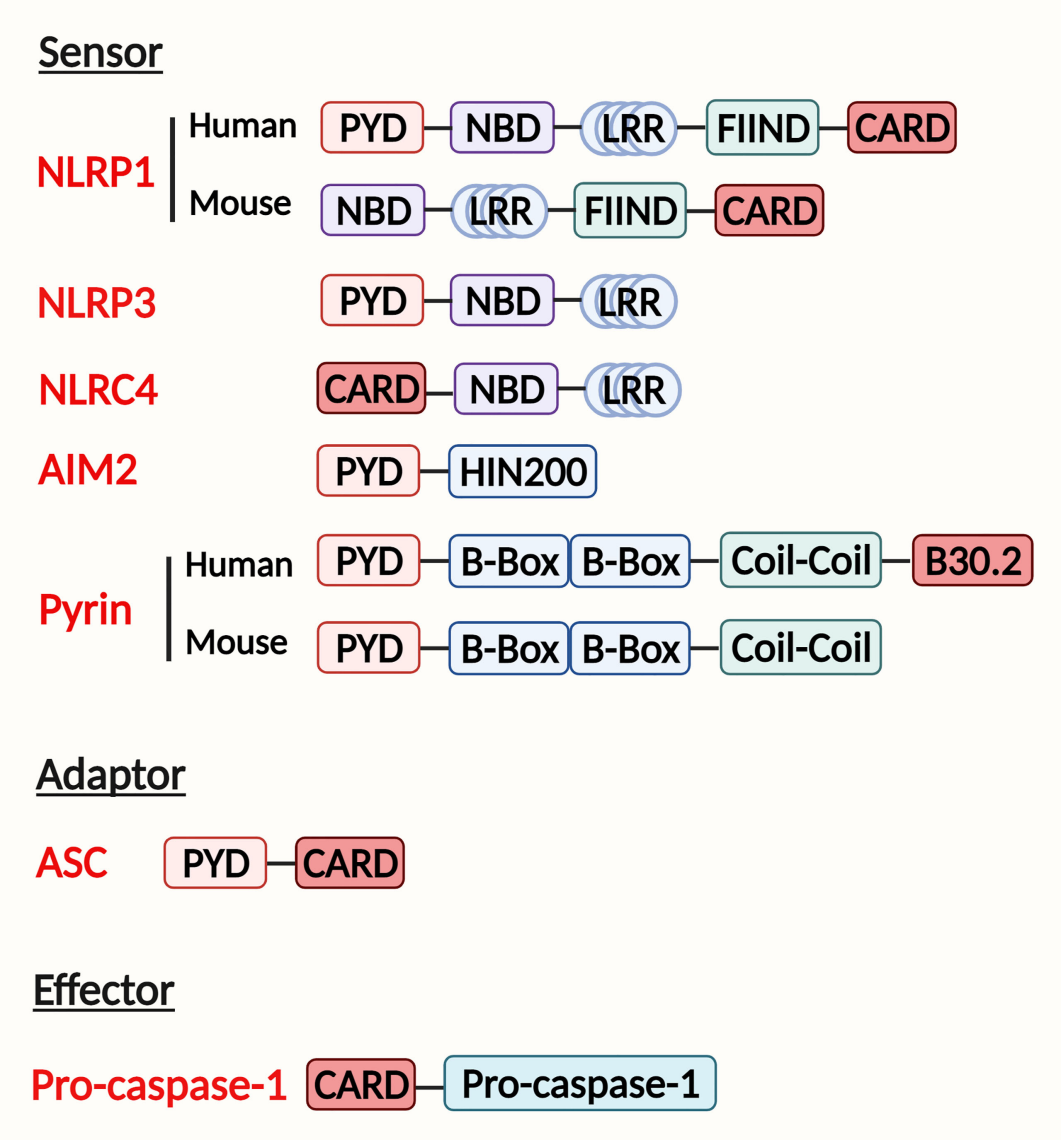
Domain structure and inflammasome assembly. The inflammasome complex contains a sensor, adapter, and effector protein. There are two kinds of sensors: the NOD-like receptor (NLR) family and the PYHIN (pyrin and HIN200 (hematopoietic interferon-inducible nuclear antigens with 200 amino-acid repeats) domain-containing protein) family. The NLR family includes NLRP1, NLRP3, NLRC4 sensors. They contain a nucleotide-binding domain (NBD), carboxy-terminal leucine-rich repeat (LRR), either a pyrin domain (PYD) or a caspase activation and recruitment domain (CARD). Additionally, NLRP1 has a function-to-find domain (FIIND) at the C terminal. The PYHIN family AIM2 receptor has a PYD and HIN200 domain, whereas pyrin contains a PYD, two B boxes, and a coil-coil domain. Human pyrin has an additional B30.2 domain. Adaptor has a PYD to combine with the sensor and a CARD with the effector. The effector is composed of a CARD and pro-caspase-1, which will be cleaved when inflammasome assembly.

There are five main kinds of inflammasome sensor complexes in terms of receptors, NLRP1, NLRP3, NLRC4, AIM2, and pyrin (**Figure 1**). The inflammasome receptor oligomerizes and recruits the adaptor ASC and effector caspase-1 to assemble inflammasome complexes after sensing pathogens or host-derived injuries. After activation, the cleaved caspase-1 mediates the maturation and secretion of IL-1β and IL-18 and pyroptotic cell death (9). In this section, we will compare the structures of different inflammasomes and discuss the mechanism of inflammasome activation.

### 2.1 Inflammasome Assembly and Structure

#### 2.1.1 NLRP1 Inflammasome

NLRP1 was the first inflammasome to be identified; however, little research has focused on NLRP1 inflammasome due to the complexity and differences in structures between humans and mice. Human NLRP1 inflammasome has two domains in NOD-like receptor (NLR): an N−terminal pyrin domain (PYD) and a C−terminal caspase activation and recruitment domain (10). In contrast, the mouse genome encodes Nlrp1a, Nlrp1b, and Nlrp1c, which activate caspase-1 without the help of an ASC adaptor due to the lack of a PYD domain in the receptor (11). But a function-to-find domain (FIIND) at the C−terminal is required in both humans and mice to participate in the activation of the NLRP1 inflammasome (**Figure 1**) (12).

Regarding inflammasome activation, Bacillus anthracis lethal toxin (LT) was involved in the NLRP1 inflammasome activation and subsequent caspase-1−dependent cytokine release and pyroptosis (13). Moreover, extracellular ATP was also demonstrated to trigger the NLRP1 inflammasome by activating the P2X7 receptor and potassium efflux (14, 15). As the NLRP1 inflammasome is less well-studied, the role of the NLRP1 inflammasome in chronic liver disease and liver fibrosis remains unclear.

#### 2.1.2 NLRP3 Inflammasome

The NLRP3 inflammasome contains the NOD-like receptor NLRP3, the adaptor ASC, and the effector pro-caspase-1 (**Figure 1**). The NLRP3 inflammasome is the most widely studied and the best-characterized inflammasome in infectious and chronic inflammatory diseases (16).

The stimuli involved in NLRP3 activation include pathogen-associated molecular patterns (PAMPs) ligands (17), such as bacterial, fungal, and viral components, pore-forming toxins, nucleic acids; and damage-associated molecular patterns (DAMPs) such as extracellular ATP (18), uric acid crystals (19), and amyloid (20). Activation by these PAMPs and DAMPs indicates that the NLRP3 inflammasome is a common sensor of cellular stress or injury. Activation of the NLRP3 inflammasome requires two steps. First, extracellular stimuli prime cells, *e.g.*, LPS binds to TLR4 to activate NF-κB and subsequent transcription and translation of IL−1β. Then secondary stimuli such as ATP induce inflammasome complex assembly and IL-1β cleavage and secretion. An increasing number of studies have confirmed the critical roles of NLRP3 inflammasome activation in pro-inflammatory cytokines release and pyroptosis initiation. The NLRP3 inflammasome-mediated cellular communication among different liver cell types in acute and chronic liver diseases from diverse liver injuries was also well-defined (21, 22), indicating the pivotal role of NLRP3 inflammasome in the development of liver diseases.

#### 2.1.3 NLRC4 Inflammasome

The NLRC4 inflammasome is activated in response to Flagellin infection (23), Salmonella infection (24), and bacterial type 3 secretion systems (T3SSs) (25). These stimulators bind to NLRC4 indirectly. During infection, neuronal apoptosis inhibitory proteins (NAIPs) interact with the ligand and NLRC4 receptor, activating the assembly of the NLRC4 inflammasome (**Figure 1**) (26). A few studies showed that bacterial flagellin induced NLRC4 inflammasome activation in hepatocytes and KCs (27, 28). Moreover, the NLRC4-driven IL-1 release was also involved in liver fibrosis (27, 28). Though these studies show an important role of NLRC4 inflammasome in bacterial infection in liver cells, more studies are needed to explore the potential mechanisms of NLRC4 inflammasome activation and its role in liver fibrosis.

#### 2.1.4 AIM2 Inflammasome

The AIM2 inflammasome is a cytosolic receptor that senses double-stranded DNA (dsDNA). It contains an N-terminal PYD domain and a C-terminal hematopoietic interferon-inducible nuclear protein with a 200-amino acid repeat (HIN200) domain (**Figure 1**) (29). When dsDNA binds to the AIM2 receptor, ASC and pro-caspase-1 are recruited for inflammasome complex formation and activation (30, 31). Previous studies have found that the AIM2 inflammasome induction in hepatocytes and macrophages participates in chronic liver diseases *via* the pyroptosis pathway (32–35). However, the mechanism of AIM2 inflammasome regulation is not well-studied.

#### 2.1.5 Pyrin Inflammasome

Pyrin, encoded by the MEFV gene, contains a PYD, two B−boxes, a coiled-coil domain, and a SPRY/PRY domain (**Figure 1**). Pyrin was recently recognized as an inflammasome-forming and pyroptosis-initiating protein. Pyrin inflammasome was reported to stimulate pyroptosis, but there is no evidence showing its direct effect on the progression of liver fibrosis (36, 37).

### 2.2 Functions of Effector Components in Inflammasomes

Inflammasome assembly activates caspase-1, which cleaves the pro-IL-1β and pro-IL-18 into active IL-1β and IL-18. Meanwhile, GSDMD is cleaved by caspase-1 to N-terminal fragments (GSDMD-NT), which are inserted into the cell lipid membrane to assemble arc‐ and slit‐like oligomers and grow into large and stable ring‐like oligomers to form transmembrane pores. This process leads to cell membrane rupture and resultant pyroptosis (38). We next explored the functions of these effector components in inflammasomes.

#### 2.2.1 IL Maturation and Secretion

Activation of inflammasome complexes cleaves pro-caspase-1 and triggers IL-1β and IL-18 maturation and secretion. IL−1β and IL−18 are crucial cytokines involved in immune responses and trigger the inflammatory cascade (39). However, there have been many debates about how IL-1 is secreted out of cells. Recent studies have shown that *Gsdmd* knockout macrophages mature IL−1β normally but fail to secrete it due to lack of pyroptosis, suggesting that IL-1β is secreted through cell membrane rupture and lysis (40). However, whether IL-1 is released *via* pyroptosis in other cell types remains unknown; further studies are needed.

#### 2.2.2 Pyroptosis

Pyroptosis is a distinct type of programmed cell death characterized by the formation of cell membrane pores, the release of intracellular contents, nuclear condensation, and cell lysis (41). Compared with pyroptosis, apoptosis and necroptosis are caspase-3/7 and receptor-interacting serine-threonine kinase-1/3 (RIPK-1/3) mediated programmed cell death, respectively (42). Pyroptosis depends on inflammatory caspase-1 to cleave gasdermins to form membrane pores. Of all gasdermins, GSDMD has been demonstrated to play a central role in pyroptotic cell death. Active caspases cleave GSDMD to GSDMD-NT, which causes membrane pores and induces pyroptosis *via* both canonical and non-canonical signaling pathways (41, 43). However, the mechanism by which GSDMD executes cell death is poorly explored.

### 2.3 Canonical and Non-Canonical Signaling Pathways of Inflammasome Activation

**Figure 2:**
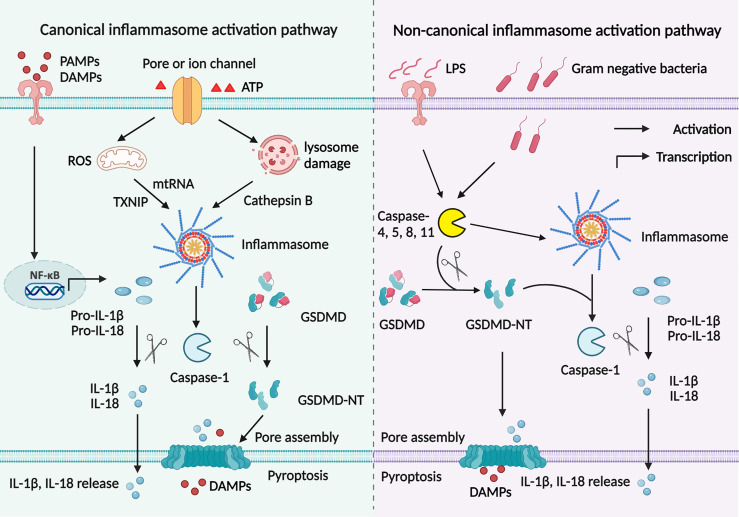
Canonical and non-canonical signaling pathways to activate inflammasomes. Canonical signaling pathway to activate inflammasomes is mediated by two signaling steps. The priming step involves PAMPs or DAMPs binding to TLRs to initiate NF- κB signaling pathway, which promotes pro-IL-1β and pro-IL-18 production. Then endogenous danger signals activate inflammasome assembly and caspase-1 in the second step. Active caspase-1 cleaves GSDMD into GSDMD-NT; cut pro-IL-1β and pro-IL-18 into mature IL-1β and IL-18. The GSDMD breaks a pore on the cell membrane to help IL-1β and IL-18 secretion. There are three main mechanisms involved in inflammasome activation. Extracellular ATP binds to the P2X7 receptor and causes potassium efflux and NLRP3 activation. Uric acid and amyloid are endocytosed in a lysosome-dependent manner, promoting lysosome rupture and cathepsin B release and resultant inflammasome assembly. TXNIP and mtDNA accumulation in a ROS-dependent manner activates inflammasomes. However, in the non-canonical signaling pathway, intracellular LPS or toxin initiates caspase-11, promoting inflammasomes assembly and caspase-1 activation. Then both active caspase-11 and caspase-1 cleave GSDMD into GSDMD-NT, leading to cascaded reactions similar to the canonical signaling pathway.

As previously reviewed, inflammasomes are initiated and assembled *via* a canonical or non-canonical signaling pathway (2). There are two steps to process inflammasome activation in the canonical signaling pathway (**Figure 2**) (44). In the first step, PAMPs, such as LPS, bind to TLRs and elicit the downstream MyD88–NF-κB signaling pathway to produce pro-IL-1β and pro-IL-18. In the second step, endogenous danger signals activate inflammasome assembly and caspase-1. A combination of PRR, ASC, and pro-caspase-1 helps activate caspase-1, which cleaves GSDMD into GSDMD-NT as well as cleaves pro-IL-1β and pro-IL-18 into mature IL-1β and IL-18. There are three main mechanisms involved in the second step. Firstly, extracellular ATP binds to the P2X7 receptor and opens a pore on the cell membrane through pannexin 1 protein, causing potassium efflux and NLRP3 activation (18). Secondly, uric acid crystals and amyloid endocytosed by phagosome lysosomes contribute to lysosome rupture and release of cathepsin B, which helps inflammasome assembly (19). Thirdly, thioredoxin-interacting protein (TXNIP) detached from thioredoxin and mitochondrial DNA (mtDNA) accumulation in a ROS-dependent manner activates inflammasomes (45).

Apart from the canonical signaling pathway, inflammasomes are also activated by the non-canonical signaling pathway (**Figure 2**). In this process, intracellular LPS or toxin initiates caspase-11, promoting inflammasome assembly and caspase-1 activation. Then both active caspase-11 and caspase-1 cleave GSDMD into GSDMD-NT, leading to cascaded reactions similar to the canonical signaling pathway (46). Inflammasome activation is a complex process that involves multiple proteins. It remains unclear whether the cross-talk of the canonical signaling pathway and the non-canonical signaling pathway also revokes inflammasome activation.

## 3 Inflammasome and Pyroptosis in Liver Fibrosis

**Figure 3:**
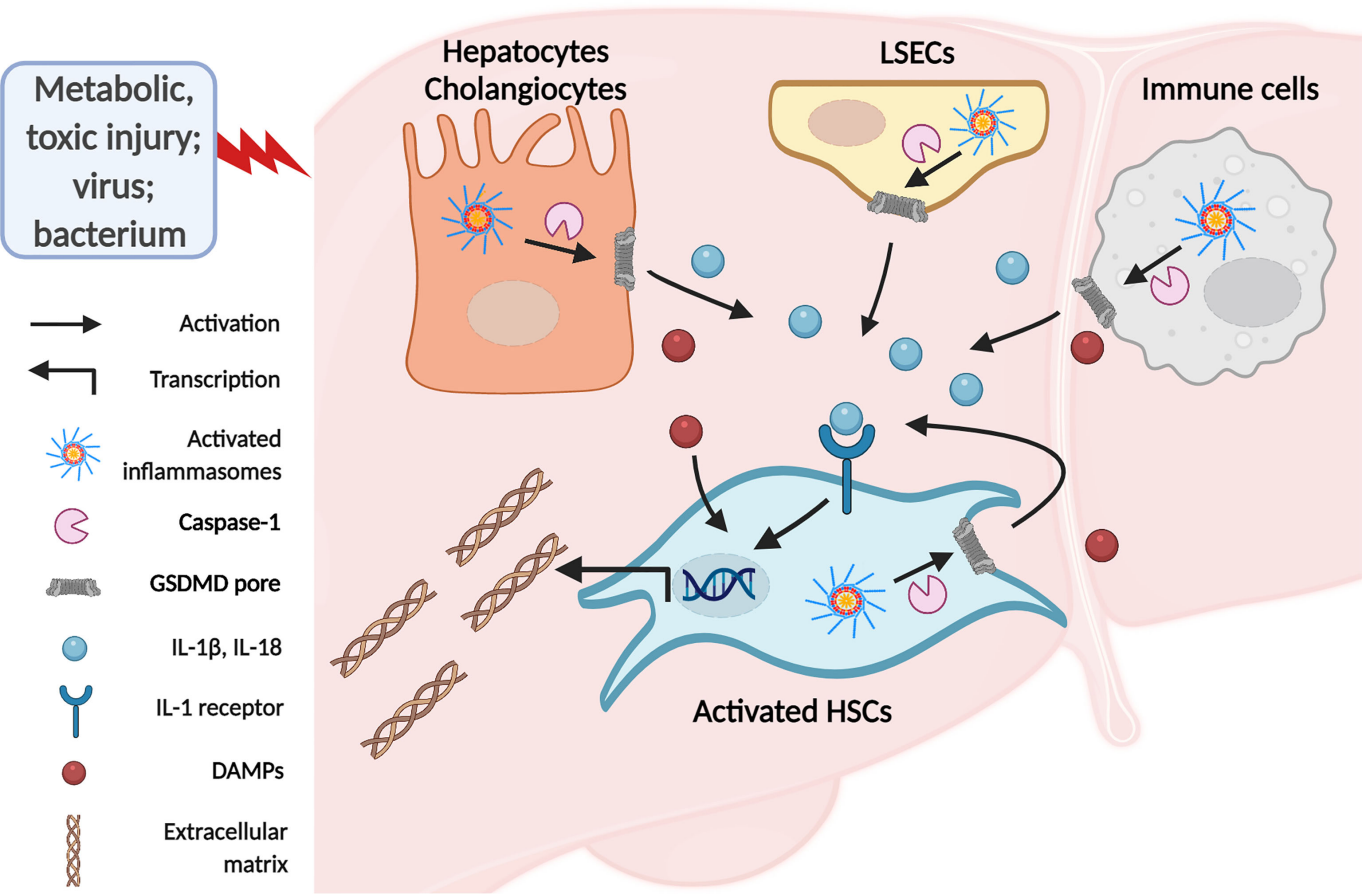
The inflammasomes in liver fibrosis. Activated HSCs are the primary source of extracellular matrix deposition in response to chronic liver injury. There are direct and indirect ways involved in inflammasome to stimulate HSCs. Activation of inflammasomes in hepatocytes and macrophages due to chronic liver injury leads to IL-1β and IL-18 release and pyroptotic cell death. Secreted IL-1β and IL-18 bind to IL-1 receptors on HSCs and induce HSCs activation and resultant ECM formation. In addition, DAMPs from pyroptotic cells promote HSCs activation and liver fibrosis. Meanwhile, PAMPs or DAMPs may directly target HSCs to induce inflammasome-dependent IL-1 release and pyroptosis, eliciting HSCs activation in an autocrine or paracrine manner.

Liver fibrosis is a dynamic process characterized by an imbalance in ECM deposition and degradation due to chronic liver injury of various etiologies (5). During this process, quiescent HSCs transdifferentiate into fibrogenic activated phenotype, representing the dominant collagen-producing myofibroblasts in different chronic injury models (5, 7, 47). Stimulators of chronic liver injuries, such as fatty acid, alcohol, toxin, and virus infection, affect hepatocytes, hepatic macrophages, LSECs, and cholangiocytes. Damaged liver cells interact with HSCs by releasing pro-inflammatory and pro-fibrotic factors, promoting HSC activation and resultant liver fibrosis (48–50). It is worth noting that inflammasome activation and pyroptosis play a central role in the inflammatory cascade (51). Previous studies have demonstrated that inflammasome components are widely expressed in these liver cell types in response to liver injury (52–54). Moreover, inflammasomes could induce liver fibrosis both directly and indirectly. Inflammasome activation in HSCs directly leads to HSCs activation, which is responsible for ECM formation and liver fibrosis. Indirectly, pro-inflammatory cytokine release or pyroptotic cell death in hepatocytes and other nonparenchymal cells induces HSC activation and eventually leads to liver fibrosis (**Figure 3**) (55).

### 3.1 Inflammasomes and Pyroptosis in Hepatocytes

Although inflammasomes are predominately expressed in immune cells, hepatocytes, which are the most abundant cell type in the liver and predisposed to liver injury, also express inflammasomes (52). Recently, increasing studies have demonstrated important roles of hepatocellular inflammasomes activation, the subsequent pyroptotic death, and inflammasome-implicated cross-talk with HSCs in chronic liver injury from various liver pathologies.

#### 3.1.1 Hepatocellular NLRP3 Inflammasome in NAFLD and NASH-Related Liver Fibrosis

Hepatocellular NLRP3 inflammasome activation and caspase-1-mediated pyroptosis play a crucial role in the progression of non-alcoholic fatty liver disease (NAFLD). Lipid exposure resulted in hepatocellular NLRP3 inflammasome activation and cleavage of mature IL-1β and IL-18 by caspase-1 in high fructose-induced NAFLD mouse model. The inflammatory response aggravated hepatocellular lipid accumulation as well as impaired insulin sensitivity in JAK/STAT3-dependent (56) and PI3K/AKT-dependent manner (57), respectively. The evidence indicates that NLRP3 inflammasome mediates lipid and glucose metabolism in hepatocytes. Mechanically, NLRP3 inflammasome and cytokine production are induced by mitochondrial reactive oxygen species (ROS) in fatty hepatocytes (58, 59). Furthermore, the binding of IL-1β and IL-18 to HSC-derived IL-1 receptor, as well as DAMPs released from pyroptotic hepatocytes transdifferentiated HSCs into activated phenotype for fibrotic scar formation (57). Liver inflammation and ECM deposition are relieved in hepatocyte-specific *caspase-11*-deficient mice with high sucrose and high fat diet, where the production of IL-1β and GSDMD was blocked, further proving the role of inflammasomes in liver fibrosis (60). In summary, these studies have demonstrated that hepatocellular NLRP3 inflammasome and pyroptosis contribute to the development of NAFLD and liver fibrosis *via* the cross-talk between hepatocytes and HSCs.

#### 3.1.2 Hepatocellular NLRP3 Inflammasome in Liver Fibrosis

Hepatocyte pyroptosis and the release of inflammasome particles caused by DAMPs and PAMPs were internalized by HSCs and further induced HSC activation and liver fibrosis (55, 61). It seems that the NLRP3 inflammasome in hepatocytes may play a pivotal role in developing liver fibrosis. Ethanol was shown to trigger hepatocyte NLRP3 inflammasome-dependent IL-1β production and pyroptosis by TXNIP overexpression (62) and the caspase-4/11–GSDMD non-canonical signaling pathway in ALD (63). Elevated inflammation accompanied apoptotic and necroptotic cell death in the *Nlrp3*-knockout acute BDL model. In contrast, decreased liver injury and bridge fibrosis in the *Nlrp3*-knockout chronic BDL model showed diverse roles of NLRP3 in acute and chronic cholestatic liver injury (64). Mechanically, endoplasmic reticulum (ER) stress is the central process involved in NLRP3 inflammasome activation in hepatocytes. The IRE1α–sXBP1–ER stress signaling pathway mediated hepatocellular NLRP3 inflammasome activation (65). In comparison, this NLRP3 inflammasome activation was relieved in XBP1 knockout mice (65). In the LPS- and CCl_4_-induced chronic liver injury model, NLRP3 inflammasome-mediated hepatocyte pyroptotic cell death was relieved after ER stress abrogation *via* either CHOP knockdown (66) or FXR regulation (67). The above studies demonstrate that ER stress-dependent inflammasome activation mediates chronic liver injury and fibrosis. However, further studies that use hepatocyte-specific NLRP3 deletion mice are urgently needed. Although many studies have revealed that NLPR3 is upregulated in hepatocytes, few studies have focused on the transcriptional regulation of NLRP3.

#### 3.1.3 Other Hepatocellular Inflammasomes in Liver Fibrosis

In addition to NLRP3, other inflammasomes in hepatocytes are also involved in liver injury and liver fibrosis. AIM2 inflammasome activation by mtDNA resulted in increased expression of IL-1β and GSDMD in hepatocytes. The release of IL-1β promoted liver steatosis, inflammation, and activated IL-1 receptor on HSCs, contributing to the fibrogenic phenotype of HSCs (32); whereas HMGB1 redox status inhibited activation of caspase-1 through AIM2 inflammasome in chronic liver inflammation (33). In addition, NLRC4 inflammasome activation and pro-inflammatory cytokines induced by bacterial flagellin were rescued by hepatocyte Toll-Like Receptor 5 (27). The progression of NASH was attenuated by the deletion of NLRC4 inflammasome and the subsequent decrease in hepatocyte pyroptosis (68). The above studies show hepatocytes express and activate other inflammasomes, which participate in cellular communication with HSCs to induce liver fibrosis. Compared to the NLRP3 inflammasome, the effect of other inflammasomes remains unclear, and further studies are needed.

In summary, various chronic injuries lead to inflammasome assembly in hepatocytes *via* canonical and non-canonical signaling pathways. Then inflammasomes and pyroptosis in hepatocytes initiate liver fibrosis mainly *via* HSC activation.

### 3.2 Inflammasomes and Pyroptosis in Hepatic Macrophages

There are two phenotypes of macrophages in the liver: liver resident KCs and bone marrow-derived macrophages (BMDMs). KCs are the predominant macrophages in healthy conditions, whereas BMDMs infiltrate into the liver in response to danger signals (49, 69). Macrophages are recognized as the primary source of inflammasomes and pro-inflammatory cytokines (70). Generally, inflammasome activation and the subsequent pyroptosis in macrophages promote chronic liver inflammation by IL-1 and DAMPs release, which bind to receptors on HSCs and initiate HSCs-mediated fibrosis from various etiologies.

#### 3.2.1 Hepatic Macrophage NLRP3 Inflammasome in NAFLD and NASH-Related Liver Fibrosis

In NASH models, the NLRP3 inflammasome activation in KCs or BMDMs caused inflammatory cytokine production, contributing to lipid synthesis in hepatocytes and fibrotic collagen production in HSCs (71–73). Furthermore, macrophage-specific *Nrf2* knockout aggravated NASH progression by initiating ROS and IL-1β in a yes-associated protein (YAP)–NLRP3-dependent manner (74). This finding demonstrates the role of ROS in activating the NLRP3 inflammasome in NASH. Similarly, the activation of the kinase receptor-interacting protein 1 (RIP1) in BMDMs, which induced inflammasome assembly and pyroptotic cell death, also contributed to the pathogenesis of NASH (75). Mechanically, NLRP3-mediated M1 macrophage polarization was blocked by the TGR5 signaling pathway, which further restored liver steatosis, inflammatory infiltration, and liver fibrosis (76). TLR2 and palmitic acid cooperatively stimulated the NLRP3 inflammasome in KCs and BMDMs. Then the activated NLRP3 inflammasome enhanced macrophage autophagy *via* the release of IL-1β to induce HSC activation (77, 78). Accordingly, inhibiting the NLRP3 inflammasome by TIM-4 *via* LKB1–AMPKα-mediated autophagy in macrophages suppressed the progression of NAFLD (79).

Moreover, the caspase-1–IL-1β signaling pathway in macrophages leads to lipid accumulation, inflammatory infiltration, and liver fibrosis in NASH mouse models by mediating macrophage–hepatocyte and macrophage–HSC interactions (80). However, this progression was blocked by ezetimibe administration in an autophagy-dependent manner (80). Consistently, by exposing hepatocytes and KCs to cholesterol crystals, NLRP3 inflammasome activation was demonstrated to be involved in the development of NASH (81). The above investigations confirmed the vital role of autophagy in regulating macrophage-derived inflammasome assembly in NASH. Nevertheless, the role of macrophage-derived autophagy in inducing disrupted cross-talk in sinusoids and liver fibrosis in NASH remains unclear. Further studies that focus on cellular cross-talk in the sinusoids are urgently needed.

#### 3.2.2 Hepatic Macrophage NLRP3 Inflammasome in Liver Fibrosis

NLRP3 inflammasome activation in KCs and BMDMs drove the caspase-1 signaling pathway, leading to IL-1 secretion and pyroptotic death in response to LPS-induced injury *in vivo* and *in vitro (* 82, 83). However, NLRP3 inflammasome-derived IL-1β secretion and pyroptosis in macrophages and collagens deposition were blocked by pharmaceutical inhibition or genetic knockdown of COX-2 (84). Consistently, COX-2 promotes the development of liver cirrhosis (85) by inducing ROS (86, 87) and ER stress (88), indicating that COX-2 might contribute to liver cirrhosis *via* macrophage-derived inflammasomes. However, further studies are needed to determine how COX-2 regulates the activation of macrophage-derived inflammasomes and pyroptosis of macrophages in the context of liver fibrosis.

Studies confirmed that NLRP3 activation was not only required for hepatic inflammation and fibrosis but also as an essential mediator to amplify and perpetuate programmed inflammatory pyroptotic cell death (55). LPS from the damaged gut barrier and endogenous danger signals from hepatocytes damaged by ethanol (extracellular ATP and uric acid) are responsible for macrophage recruitment and subsequent inflammatory cytokine processing and pyroptotic cell death (89–92). The process is termed classically activated (M1) macrophage polarization (93). Increased NLRP3 and subsequent IL-1β maturation and secretion in macrophages exacerbated liver inflammation and fibrosis in ALD *via* induction of IL-1 receptor expression on HSCs and facilitated ECM secretion and formation (91). However, macrophage-specific *Atg5* knockout promoted caspase-1 activation, and pro-inflammatory M1 macrophage polarization, resulting in liver fibrosis during chronic ethanol exposure. Autophagy, a conserved cellular process to remove the damaged or unnecessary component, plays an important role in negatively regulating NLRP3 inflammasome in macrophages by degrading mitochondrial-derived DAMPs and inflammasome complexes. Research has demonstrated that autophagy in macrophages is protective against alcohol-induced liver injury in an NLRP3 inflammasome-dependent manner (94). Taken together, NLRP3 inflammasome induction in macrophages polarizes M1 phenotype, which is under the negative regulation of the autophagy signaling pathway.

Patients with chronic HCV infection and cirrhosis have elevated serum Il-1β and IL-18 levels (95, 96). Increased serum IL-1β and IL-18 are the components of NLRP3 inflammasome activation in response to HCV uptake by liver macrophages (95, 96). The current results confirm that NLRP3 inflammasome assembly and activation are central to mitigating HCV-related inflammation and fibrosis (95–97). Besides, NLRP3 inflammasomes expressed in KCs cause inflammatory cytokine production and fibrotic collagen formation in schistosomiasis-induced liver fibrosis (SSLF) (98) and idiosyncratic liver injury-induced fibrosis (99, 100). The above observation confirms that the NLRP3 inflammasomes in hepatic macrophages are crucial in initiating and propagating liver fibrosis from different etiologies.

#### 3.2.3 Other Hepatic Macrophage Inflammasomes in Liver Fibrosis

Not only NLRP3 inflammasome but also other inflammasome complexes are involved in liver injury and liver fibrosis. Assembly of the AIM2 inflammasome in KCs led to the processing of IL-1β and IL-18 in response to hepatitis B virus infection, aggregating the development of liver cirrhosis (34, 101). Moreover, perfluorooctane sulfonate (PFOS), a chemical that causes chronic systematic inflammation, activated the AIM2 inflammasome *via* the Ca(2+)–PKC–NF-κB/JNK–BAX/BAK axis (35). In contrast, deletion of the AIM2 suppressed PFOS-induced inflammation and fibrosis in the liver and other organs (35). This study shows the critical role of the AIM2 inflammasome in toxin-induced chronic inflammation (35).

The NLRP6 inflammasome promotes the onset of hepatic granuloma and collagen deposition, indicating that NLRP6 is a crucial trigger for SSLF (102). Activation of the NLRC4 inflammasome and the subsequent pyroptosis and IL-18 and IL-1β secretion in macrophages promotes cross-talk with HSCs, exacerbating inflammation and fibrosis development in NAFLD (28). Furthermore, the NLRP12 inflammasome negatively modulated inflammatory responses by blocking the NF-κB and MAPK signaling pathways as well as IL-1β release from BMDMs in mouse liver and spleen against the infection of Brucella abortus, which is a kind of Gram-negative bacterium infection causing innate immunity and subsequent chronic inflammation in the host (103).

In summary, inflammasome activation mediates the polarization of liver macrophages and subsequent interaction with HSCs, contributing to the progression of liver fibrosis.

### 3.3 Inflammasomes and Pyroptosis in Cholangiocytes

Cholangiopathies, *e.g.*, primary sclerosing cholangitis (PSC) and primary biliary cholangitis (PBC), represent an autoimmune inflammatory liver disease characterized by chronic inflammation and subsequent sclerosis and destruction of intrahepatic small bile ducts (104). Increased NLRP3 expression in reactive cholangiocytes resulted in pro-inflammatory IL-18 production and influenced the epithelial integrity of cholangiocytes in both murine PSC model and patients with PSC (105). Meanwhile, inflammasome activation in cholangiocytes interacts with nuclear translocation of pSer675β-catenin and transcriptional activation, which recruits M1 macrophages in a CXC-chemokine ligand-1/10/12 (CXCL-1/10/12) dependent manner and activates HSCs in a transforming growth factor-β (TGF-β) dependent manner. These liver cell phenotype changes initiate biliary inflammation and fibrosis (106, 107). Furthermore, galectin-3, a pleiotropic lectin that mediates cell-cell adhesion, is secreted by these inflammatory macrophages and exacerbates cholangiocyte injury. While inflammasome activation and PBC-induced fibrosis can be improved by deleting macrophage-derived galectin-3 expression in the mouse model (108).

### 3.4 Inflammasomes and Pyroptosis in HSCs

In addition to cross-talk through inflammasomes between other liver cell types and HSCs, HSC-derived inflammasomes can be remarkably induced in pathological conditions. Inflammasome activation in HSCs initiates a range of functional changes such as transdifferentiating into a fibrogenic activated phenotype, as well as inhibition of chemotaxis (109).

#### 3.4.1 HSC NLRP3 Inflammasome in Liver Fibrosis

Palmitic acid upregulated the NLRP3–IL-1β axis in HSCs *via* the TLR4–MyD88–NF-κB signaling pathway (110) and hedgehog signaling pathway (111). Then the activated NLRP3 facilitates IL-1 receptor expression on HSCs and the consequent fibrotic induction with the development of NASH (111). Alternatively, the HSC-derived NLRP3 inflammasome could also be activated *via* the PDGFβR–NLRP3–caspase-1 signaling pathway (112), and result in increased expression of fibrotic markers alpha-smooth muscle actin (α-SMA), connective tissue growth factor (CTGF), and tissue inhibitors of matrix metalloproteinase 1 (TIMP1) in the CCl_4_ mouse model. Mechanically, many molecules are involved in the activation of the NLRP3 inflammasome. ROS plays a vital role in activating the NLRP3–IL-1β signaling pathway in HSCs (113). Angiotensin II-mediated NLRP3 inflammasome assembly contributed to cholestatic liver fibrosis (114), whereas *Nlrp3* knockout or inhibition of Ang- (1–7) reduced ECM synthesis and deposition (115). The vitamin D receptor (VDR) agonist calcipotriol alleviated cholestatic fibrosis *via* YAP1 mediated inactivation of the NLRP3 inflammasome and caspase-1 (116). The P2X7 receptor, which binds with endogenous danger extracellular ATP, is involved in the immune response and inflammation by activating the NLRP3 inflammasome and increasing IL-1β production and pyroptosis in HSCs during the development of chronic alcoholic liver fibrosis (117). Meanwhile, the release of inflammasome components from pyroptotic HSCs, in turn, activates quiescent HSCs (117). Cysteine–cysteine chemokine ligand 5 (CCL5) secreted from macrophages, activated HSC-derived NLRP3, IL-1β, and IL-6 and upregulated liver fibrosis markers α-SMA and TGF-β1 (118). In contrast, blocking antibodies against CCL5 inhibited HSCs activation and HCV-related liver fibrosis (118).

#### 3.4.2 HSC Inflammasomes in Infection-Related and Hormone-Related Liver Fibrosis

Brucella abortus infection not only affected macrophages but also triggered NLRP3 and AIM2 inflammasome assembly in HSCs, which eventually led to collagens deposition in mouse liver (119). Schistosoma Japonicum is a parasite that causes granulomatous inflammation and tissue damage. Infection with Schistosoma Japonicum stimulated the NLRP3 inflammasome *via* Dectin-1, and JNK signaling pathways, contributing to SSLF (120). Aldosterone, the main mineralocorticoid steroid hormone secreted by the adrenal gland, was shown to play a role in regulating myofibroblast contraction and proliferation by assembly of NLRP3 inflammasome in HSCs. NLRP3 depletion in primary mouse HSCs attenuated liver fibrosis in the presence of aldosterone, indicating the crucial role of NLRP3 inflammasome in aldosterone-mediated liver fibrosis (121).

The above research indicates that the NLRP3 inflammasome is the most well-studied inflammasome in HSCs. Whether other inflammasomes in HSCs are also involved in the development of liver fibrosis remains unclear.

### 3.5 Inflammasomes and Pyroptosis in Other Liver Cells

Apart from the abovementioned cells, inflammasome activation has also existed in other liver cells. DAMPs from fatty hepatocytes initiate NLRP3 inflammasome complex assembly *via* the P2X7 receptor on sinusoidal lining cells such as LSECs (122), resulting in NASH-associated fibrosis (123). Inflammasome activation in natural killer (NK) cells promotes HSC apoptosis and alleviates the progression of liver fibrosis in a TRAIL-dependent degranulation manner (124). In contrast, natural killer T (NKT) cells had dual roles in regulating liver fibrosis *via* activating the NLRP3 inflammasome (125). Besides, the NLRC4 inflammasome in neutrophils initiated auto-inflammatory disease, and these effects were attenuated in Asc knockout mice or after IL-1 receptor inhibitor administration (126).

The impact of the different inflammasomes in individual cells varies in liver diseases and animal models. Research that uses high-throughput technologies might help us establish the landscape of inflammasomes in liver cells cross-talk. Additionally, more attention should be paid to the mechanism of inflammasome-induced pyroptosis of individual liver cells during different pathological stimulations.

## 4 Inflammasome-Targeting Therapies in Liver Fibrosis

An increasing number of studies have confirmed the involvement of inflammasomes in the development of chronic inflammation-induced liver fibrosis, indicating the possibility of therapies targeting inflammasome complex activation as well as signaling pathways involved in IL-1β and IL-18. Here, we summarized the pharmacological therapies targeting inflammasome- and pyroptosis-related signaling pathways on chronic liver injury-induced fibrosis from clinical trials and pre-clinical experimental studies (**Table 1**).

**Table 1:** Potential Therapeutic Agents for liver fibrosis.

Targeting molecule	Therapeutic agent	Targeted disease	Species	Reference
IL-1 receptor Inhibitor	Anakinra	ALD, Diabetes; Toxin-induced liver fibrosis	Human; Mouse	(55, 91, 127–129)
IL-1 inhibitor	Canakinumab	ALD, CVD	Human	(122)
Caspase-1 inhibitor	Ac-YVAD	NASH associated fibrosis	Mouse	(130)
	Vx-166 (pan-caspases)	NASH associated fibrosis	Mouse	(131)
	Emricasan (pan-caspases)	NASH associated fibrosis	Mouse	(132)
NLRP3 inhibitor	MCC950	NASH associated fibrosis	Mouse	(133)
	P2X7R	Toxin-induced liver fibrosis; NASH associated fibrosis	Mouse; Human cell	(18, 134)

### 4.1 IL-1 Inhibitors

Macrophages, hepatocytes, and HSCs are the major cells that produce inflammasome-driven IL-1β due to ethanol and LPS stimulation. Recombinant IL-1R antagonist administration in mice inhibited the IL-1 signaling pathway and reversed alcohol-induced liver steatosis, inflammation, and fibrosis (91, 129). Anakinra, a recombinant form of human IL-1R antagonist, is FDA approved medicine to treat rheumatoid arthritis and neonatal-onset multisystem inflammatory disease (135). It was shown that Anakinra could relieve macrophage infiltration, lipid accumulation, and liver fibrosis in a mouse model of ALD (129). Moreover, Anakinra treatment alleviated liver injury and inflammation without affecting fibrosis in a mouse model of CCl_4_-induced liver fibrosis, as shown by a decrease in caspase-1 and IL-1β with unchanged CTGF and TIMP expression (55). Since good efficiency in treating experimental liver diseases, Anakinra combined with zinc and pentoxifylline is used to treat patients with severe alcoholic hepatitis and type 2 diabetes. Unfortunately, Anakinra did not affect 30-day mortality in research focused on 3-month and 6-month survival rates (ClinicalTrials.gov, AH/NCT01809132) (127). In another clinical trial, Anakinra was administered to patients with type 2 diabetes. They found a decrease in insulin resistance and systematic inflammation, which also participate in the progression of NASH (128). Further clinical trials on Anakinra in patients with NASH are needed. Canakinumab, a monoclonal antibody against IL-1β, was beneficial in patients with cardiovascular diseases (122), and a clinical trial about its effect on patients with severe alcoholic hepatitis is ongoing (ClinicalTrials.gov, AH/NCT03775109). Although many basic studies have shown beneficial effects by inhibiting the IL-1 singling pathway, few have been translated into clinical treatment of chronic liver diseases and liver fibrosis. More clinical trials are needed to explore the role of IL-1 in liver fibrosis.

### 4.2 Caspase-1 Inhibitors

The pan-caspase inhibitors, Vx-166 and Emricasan, showed a beneficial role in liver inflammation and fibrosis in NASH mice by decreasing the expression of IL-1β and IL-18 and inactivating HSCs (131, 132). A caspase-1 specific inhibitor, Ac-YVAD, was demonstrated to block liver steatosis and fibrosis in mice fed with HFD (130). In view of the excellent effect on animal models, clinical trials of caspase-1 inhibitors on chronic liver diseases are urgently needed in the future.

### 4.3 Inflammasomes and Their Upstream Inhibitors

The NLRP3 inflammasome inhibitor MCC950 was reported to relieve liver inflammation *via* polarizing macrophages into M2 phenotype in CCl_4_-induced liver injury (136) and fibrosis *via* decreasing plasma and hepatic IL-1β and IL-6 in a mouse model of NASH (133), suggesting the pivotal role of MCC950 in attenuating liver inflammation from various etiologies. As the upstream molecule of the inflammasome, the P2X7 receptor induces inflammasome activation (18, 117). P2X7 receptor pharmacological inhibitor SGM-1019 was shown to block IL-1β secretion in KCs, HSC activation, and collagen deposition in human cells from NASH and in the primate model from CCl_4_-induced liver fibrosis (134). Although inflammasome inhibitors showed promising effects in experimental models, whether these inhibitors could improve liver inflammation and fibrosis in patients with liver cirrhosis remains obscure.

## 5 Future Perspectives

Inflammasome, a double-edged sword in liver injury, could protect the liver from pathogen infection, metabolism syndrome, and oxidative stress by eliminating the initial cause of cell injury and promoting wound healing. However, excessive and chronic inflammasome activation contributes to the pathogenesis of various liver diseases, which is the primary topic of our review. In summary, the pathological role of inflammasomes in liver fibrosis has gained substantial recognition from diverse chronic liver injury models. Exogenous and endogenous danger signals activate inflammasomes *via* canonical or non-canonical signaling pathways, leading to increased IL-1β, IL-18, and pyroptotic cell death. *In vivo* and *in vitro* studies over the recent years have evidenced central roles of IL-1β, IL-18, and pyroptotic cell death in NLRP3 inflammasome-induced biological responses, but the functions of the non-canonical inflammasome activation signaling pathway and other NLR genes in liver fibrosis remains poorly understood. Further studies are needed to explore the cellular source of other inflammasomes and their roles in liver fibrosis. In addition, the mechanism of GSDMD assembly leading to cell lysis is largely unknown. Apart from IL-1, inflammasomes also regulate the release of other immune factors, such as leukotrienes and prostaglandins (137), which could regulate liver fibrosis progression (85). Therefore, further studies are required to explore the potential mechanisms.

Quiescent HSCs change phenotypes and have different cell fates after stimuli, including activation, senescence, and inactivation. These phenotypes can be transdifferentiated from each other (5). Most studies illustrate that HSCs are activated by paracrine profibrogenic cytokines and danger signals, while other molecules from pyroptotic cells that regulate HSC fate have scarcely been investigated. Specifically, it remains unclear whether inhibition of inflammasome-mediated cell interactions with HSCs could regress liver fibrosis. Although IL-1 from inflammatory hepatic macrophages could activate HSCs, the release of matrix metalloproteinases (MMPs) from these inflammatory macrophages degrades ECM and resolves fibrosis (138). Inflammasome-activated NK cells and NKT cells secreted inflammatory cytokine IL-1 to promote liver fibrogenesis, whereas IFN-γ secreted from the two kinds of cells have an antifibrotic role and trigger HSC apoptosis, promoting the regression of liver fibrosis (139, 140). Therefore, the cell-specific mechanisms of intercellular cross-talk in HSC activation need in-depth investigation.

In recent years, the pharmacological treatment of inflammasomes in chronic liver injury mainly focuses on IL-1β and IL-1 receptors. Research on blocking other molecules in the inflammasome pathway, such as caspase-1 and GSDMD, is missing. Though a recent study has shown that IL-18 inhibitor attenuates renal fibrosis in the ischemia-reperfusion murine model (141), there is no research on IL-18 inhibitors treating liver fibrosis in either animal models or human. Further studies are urgent to explore the potential of blocking IL-18, as well as caspase-1 and GSDMD in treating liver fibrosis. Although inflammasomes have been confirmed to have critical effects in many experimental animal models and *in vitro cell lines*, no direct evidence demonstrates that inflammasomes mediate liver fibrosis in humans. Organoids are self-organized 3D tissue cultures from stem cells that could recapitulate the function of the represented organ (142). Hence, organoids may be a novel research method to explore the mechanism of the inflammasome in regulating liver fibrosis. It is shown that bone marrow-derived mesenchymal stem cells (BM-MSCs) injection could decrease inflammasomes in the ischemic stroke rat model (143). Therefore, cell therapy by *in vivo* delivery of BM-MSCs may be helpful to mitigate inflammasomes during the development of liver fibrosis. Further studies are needed to target the novel direction. In summary, targeting inflammasome signaling pathways to treat liver fibrosis is promising, and more basic research and clinical trials are demanded in the future.

## Author Contributions

CT and JG designed and coordinated the study. CG, QC, CT, and JG analyzed articles and finalized the figures. CG, QC, and JG wrote the manuscript. All authors contributed to the article and approved the submitted version.

## Funding

This work was supported by the National Natural Science Fund of China (82170623, 82170625, U1702281, 81873584, 82000613, and 82000574), National Key R&D Program of China (2017YFA0205404), Sichuan Science and Technology Program (2020YJ0084 and 2021YFS0147), and the 135 projects for disciplines of excellence of West China Hospital, Sichuan University (ZYGD18004).

## Conflict of Interest

The authors declare that the research was conducted in the absence of any commercial or financial relationships that could be construed as a potential conflict of interest.

## Publisher’s Note

All claims expressed in this article are solely those of the authors and do not necessarily represent those of their affiliated organizations, or those of the publisher, the editors and the reviewers. Any product that may be evaluated in this article, or claim that may be made by its manufacturer, is not guaranteed or endorsed by the publisher.
